# A Rapid Robust Method for Subgrouping Non-NF2 Meningiomas According to Genotype and Detection of Lower Levels of M2 Macrophages in *AKT1 E17K* Mutated Tumours

**DOI:** 10.3390/ijms21041273

**Published:** 2020-02-13

**Authors:** Claire L. Adams, Emanuela Ercolano, Sara Ferluga, Agbolahan Sofela, Foram Dave, Caterina Negroni, Kathreena M. Kurian, David A. Hilton, C. Oliver Hanemann

**Affiliations:** 1 Faculty of Health: Medicine, Dentistry and Human Sciences, The Institute of Translational and Stratified Medicine, University of Plymouth, The John Bull Building, Plymouth Science Park, Research Way, Plymouth PL6 8BU, UKcaterina.negroni@plymouth.ac.uk (C.N.); 2 Department of Neurosurgery, University Hospitals Plymouth NHS Trust, Derriford Road, Plymouth PL6 8DH, UK; 3 Institute of Clinical Neuroscience, University of Bristol and Southmead Hospital, North Bristol Trust, Bristol BS8 1QU, UK; 4 Cellular and Anatomical Pathology, University Hospitals Plymouth NHS Trust, Derriford Road, Plymouth PL6 8DH, UK

**Keywords:** meningioma, M2 macrophage, genotype, *AKT1 E17K*, NF2, non-NF2

## Abstract

The majority of meningiomas are grade I, but some grade I tumours are clinically more aggressive. Recent advances in the genetic study of meningiomas has allowed investigation into the influence of genetics on the tumour microenvironment, which is important for tumorigenesis. We have established that the endpoint genotyping method Kompetitive Allele Specific PCR (KASP™) is a fast, reliable method for the screening of meningioma samples into different non-NF2 mutational groups using a standard real-time PCR instrument. This genotyping method and four-colour flow cytometry has enabled us to assess the variability in the largest immune cell infiltrate population, M2 macrophages (CD45^+^HLA-DR^+^CD14^+^CD163^+^) in 42 meningioma samples, and to suggest that underlying genetics is relevant. Further immunohistochemistry analysis comparing *AKT1 E17K* mutants to WHO grade I NF2-negative samples showed significantly lower levels of CD163-positive activated M2 macrophages in meningiomas with mutated *AKT1 E17K,* signifying a more immunosuppressive tumour microenvironment in NF2 meningiomas. Our data suggested that underlying tumour genetics play a part in the development of the immune composition of the tumour microenvironment. Stratifying meningiomas by mutational status and correlating this with their cellular composition will aid in the development of new immunotherapies for patients.

## 1. Introduction

Meningiomas arise from the meningeal layers surrounding the brain and spinal cord and are the most common primary intracranial tumours, accounting for 36% of all primary central nervous system tumours [1]. Although 70–80% of these tumours are considered to be benign (WHO grade I), 15–20% atypical (WHO grade II) and approximately 5% anaplastic (WHO grade III), a significant proportion of patients with any of the three meningioma grades will eventually relapse [2]. The histological grading of meningiomas can be falsely reassuring as there can be discordance between the histology and clinical behaviour, with some grade I tumours behaving in a more clinically aggressive manner. Combining recent genetic findings, including DNA methylation data [3], could lead to a potential molecular classification that will be better at predicting patient outcomes [4,5]. Surgical resection with adjuvant radiotherapy in grade II and III tumours (higher-grade meningiomas) remains the primary treatment option, with no clinically efficacious drug treatment for the management of meningiomas currently known [6].

Mutations or deletions on the Neurofibromin 2 (*NF2*) gene, which is located on the 22q12.2 locus and encodes the tumour suppressor Merlin [7], cause approximately half of all sporadic meningiomas [8]. NF2 gene inactivation can occur with mutations in other genes on chromosome 22, such as the SW1/SNF chromatin remodelling complex subunit, SMARCB1 [9,10,11], and it is frequently mutated in higher grades with associated worse prognosis [12]. Meningiomas with non-mutated *NF2* genes (collectively known as non-NF2 meningiomas) have been reported to be caused by somatic driver mutations in genes associated with tumorigenesis, such as Tumour necrosis factor receptor associated factor (*TRAF7*), Krupple like factor 4 (*KLF4*), v-akt murine thymoma viral oncogene (*AKT1*) and smoothened (*SMO*) [9,13]. Moreover, next-generation sequencing has also detected mutations in *POLR2A, BAP1, PIK3CA, AKT3* and *FGFR3* [11,14,15,16]. These non-NF2 meningiomas represent approximately 40% of mainly grade I sporadic meningiomas, with the other 15–20% of meningiomas containing presently unknown genetic drivers of tumorigenesis [8,17]. Interestingly, meningioma mutations have been shown to correlate with specific histological subtypes and anatomical location; however, the functional effects of these point mutations on the tumour microenvironment are unknown. Meningiomas that occur due to *NF2* mutations tend to be transitional or fibroblastic, and are located in the convexity or (lateral or posterior) skull base, whereas non-NF2 meningiomas are more medially located [18]. For example, *AKT1*-mutated tumours are found in the anterior skull base, are usually histologically meningothelial or transitional in nature, and tend to recur more quickly [19]. Interestingly, *NF2* mutations are enriched in higher-grade tumours [20].

The literature does suggest a more immunosuppressive environment in higher-grade meningiomas [21,22,23]. Macrophages are the most abundant immune cell in the meningioma microenvironment, with lower variable percentages of other immune filtrates such as T-, B- and NK cells [24,25]. Macrophage numbers are higher in grades II and III [26,27], and greater numbers have been associated with monosomy 22/del(22q) karyotype [21]. Although activated M2 macrophages have not been assessed in meningiomas, other brain tumours such as gliomas [28], glioblastomas [29] and numerous other disease models have been shown to produce signature cytokines such as TNF-alpha, TGF-beta, IL-6 and IL-10 [30,31,32].

The oncogene *AKT1* has only one known hotspot, *AKT1 E17K* (c.49G>A; p.Glu17Lys), where the mutation in the pleckstrin homology domain causes constitutive AKT1 activation, enhancing cell proliferation and tumour growth [33]. This mutation is present in 8% of all meningiomas, but is also observed in numerous other solid tumours, including approximately 3.5% of breast cancers, 3% of endometrial cancers and 1.5% of ovarian cancers (COSMIC database v90 [34]). To our knowledge, macrophage populations in *AKT1 E17K*^-^mutated meningiomas and other cancers have not been studied to date, although it is known that the PI3K/AKT/mTOR pathway regulates macrophage biology [35].

In this study, we screened 171 meningiomas using endpoint genotyping and linked mutations in *AKT1 E17K* and *NF2* in grade I meningiomas to macrophage infilitration using four-colour flow cytometry and immunohistochemistry. Our data suggest that the underlying genetics of the tumours play a part in development of the immune composition of the tumour microenvironment.

## 2. Results

### 2.1. Mutational Hotspots were Detected at the Predicted Frequencies

We screened fresh meningioma samples prospectively using an endpoint genotyping method to aid the stratification of our research into different genetic subgroups. Kompetitive Allele Specific PCR (KASP™) is a fluorescence-based genotyping method which can bi-allelically score any known single-point DNA variant and small insertions or deletions (indels) using a standard real-time PCR instrument available in most research and clinical laboratories.

A total of 171 meningiomas were screened using a KASP™ genotyping panel containing the following somatic coding mutations: *AKT1 E17K, KLF4 K409Q, PIK3CA E545K, PIK3CA H1047R, POLR2A Q403K, POLR2A L438-H439del, SMARCB1 R374Q, SMARCB1 R377H, SMO L412F* and SMO W535L (Table 1). Due to the complex nature of NF2 loss by pathogenic single mutations occurring across all exons or partial/complete deletions of the *NF2* gene, KASP™ genotyping was not suitable. Next-generation sequencing and multiplex ligation-dependent probe amplification were used on a small number of tumours to validate loss of the Merlin (NF2) protein by Western blotting [36]. NF2 status was then assessed by either intact Merlin protein (non-NF2 meningiomas) or Merlin loss (NF2 meningiomas).

Sanger sequencing was used to validate heterozygous mutations in the *AKT1* and *KLF4* genes identified by KASP genotyping (Supplementary Table S1). We also confirmed by sequencing that all *AKT1 E17K* mutants tested contained co-mutations in the WD40 region of the *TRAF7* gene. Our genotyping data displayed similar mutational frequencies to those described by Clark et al. in 2016 [11]. The slight differences in frequency may have been a reflection of our relatively small sample size (Table 1). Half of our non-NF2 meningiomas (NF2-positive by Western blotting) contained no detectable mutations according to our genotyping screening, and further in-depth sequencing analysis is required, which is in agreement with the published literature [8].

### 2.2. Correlation of Genomic Subgroup with Clinical Characteristics

In agreement with current literature, there was a higher incidence of meningioma in female patients (76%), but 30% of the male patients harboured the more aggressive form of the tumour [20]. The median age of the patients in this study was 59 years. Previous publications have described associations between driver mutations for meningiomas, their intracranial location and histological subtype. These include the propensity for NF2 tumours to be located in the posterior fossa while non-NF2 tumours tend to be located in the anterior or middle cranial fossae, and *AKT1 E17K/TRAF7*-mutant meningiomas commonly being of meningothelial or transitional histological subtypes while the *KLF4-K409Q* mutant tumours are often of the secretory subtype. In our dataset of tumours, all the *SMO L412F* mutant tumours were observed exclusively as convexity supratentorial tumours and meningothelial subtypes, while the *PIK3CA H1047R* mutant tumours were sphenoid wing meningiomas. The vast majority of our non-NF2 mutant tumours occurred in female patients, and all these tumours were WHO grade I tumours (Supplementary Table S1). In summary, our results confirmed that KASP™ genotyping is a cheap, fast, robust method for the detection of known common non-NF2 mutations.

### 2.3. Detection of M2 Macrophages by Four-Colour Flow Cytometry

Domingues et al. [26] clearly demonstrated that it is possible to detect tumour-associated macrophages in single-cell suspensions using mechanical disaggregation by multicolour flow cytometric immunophenotyping, and we therefore decided to use this technique to quantify the immune infiltrates in our well established primary cell culture model [37] and to correlate these results to mutational hotspots.

**Figure 1:**
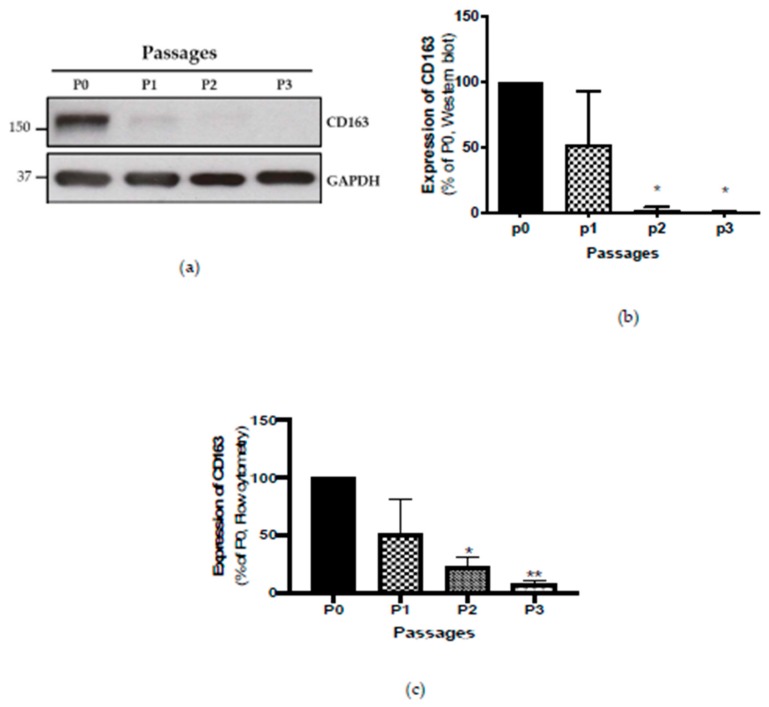
(**a**) Representative Western blot image of expression of CD163 over Passages 0 to 3 (**b**) Bar represents average value of CD163 expression by Western blot normalised to Passage 0 (p0, *n* = 3) (**c**) Bar represents average value of CD163 expression by flow cytometry normalised to Passage 0 (*n* = 3), * *p* ≤ 0.05, ** *p* ≤ 0.01.

Initially, we assessed the CD4^+^ T cells, CD8^+^ T cells, CD19^+^ B cells, and CD14^+^ monocytes within the CD45^+^ lymphocyte population. Although the percentage of CD45^+^ immune cells varied noticeably in individual tumours, CD14^+^ monocyte cells were the most dominant population within all tumours, representing over 90% of the CD45-positive immune infiltrate in samples tested (Supplementary Table S2). Using the M2 macrophage marker CD163, we determined that the majority of the CD14^+^ monocytes were CD163-positive. We showed that CD163 protein expression was high in Passage 0 by Western blotting, and this decreased over passages (Figure 1). In accordance with these data, flow cytometry results also showed a decrease in the percentage of CD45^+^HLA-DR^+^CD14^+^CD163^+^ cells by a similar amount during subsequent culture passages (Figure 1; Supplementary Figure S1).

**Figure 2:**
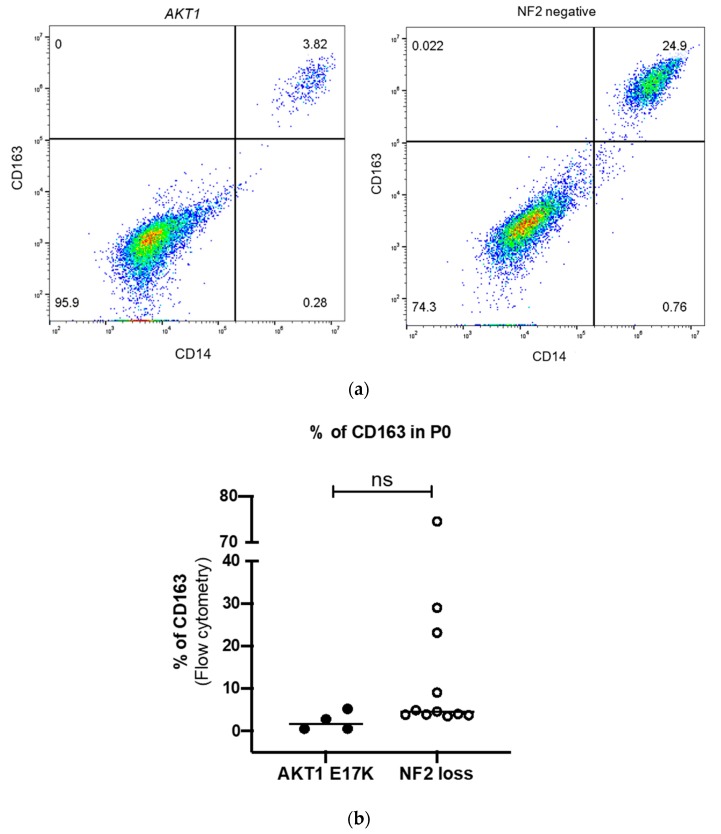
(**a**) Representative flow cytometry dot plot for CD14-PerCP-Cy5.5+ (FL3) CD163-AF647+ (FL4) cells in *AKT1 E17K* and NF2-negative primary cell culture at Passage 0 (p0) (**b**) Comparison of the CD45^+^HLA-DR^+^CD14^+^CD163^+^ M2 macrophages in *AKT1 E17K* mutants (*n* = 4) and WHO grade I NF2 negative meningiomas (*n* = 11). Line represents the median, ns = not significant.

Next, we analysed 42 meningioma samples for both M2 macrophages (CD45^+^HLA-DR^+^CD14^+^CD163^+^) and tumour cells (CD45^−^HLA−DR^−^CD14^−^CD44^+^) at Passage 0, and related these results to mutational status. The percentage of M2 macrophages was very variable from 0% to 75% with a median of 5%. These results were complemented by assessing the percentage of tumour cells at the same time point, which varied from 25% to 97% with a median of 88.7% (Supplementary Table S2). Focussing on WHO grade I meningiomas with different defined genotypes, NF2 meningiomas displayed a tendency towards higher M2 macrophage infiltrates when compared to *AKT1 E17K*-mutated tumours (Figure 2).

### 2.4. Confirmation of Macrophage Levels Using Immunohistochemistry

Next, we decided to confirm by immunohistochemistry the flow cytometry results at Passage 0, which showed that genotype could have some influence on the immune microenvironment (IHC). In total, we carried out staining on 20 meningiomas (11 non-NF2 vs. 9 NF2) using the macrophage markers CD68 (pan), CD86 (M1), CD163 (M2) and CD206 (M2). In general, the CD86 M1 macrophage marker was of weak staining intensity in all non-NF2 and NF2 cases. CD163-positive M2 macrophages were identified in all cases and at variable levels (range: 3.5–23.1%; median: 9.7%) confirming our flow cytometry data. Both methods were carried out in the same tumours, but the percentage of CD163-positive cells detected by four-colour flow cytometry analysis did not correlate directly to the number detected by IHC. The percentage of CD163-positive cells detected by IHC related well with the pan-macrophage marker CD68 (range: 2.3–18.5%; median: 9.4%) and another M2 macrophage marker, CD206 (Supplementary Table S2). These results were in agreement with recently published data reporting that the majority of tissue-associated macrophages in meningiomas are M2 macrophages with high CD163/CD206 staining and low CD80/CD86 staining [27].

**Figure 3:**
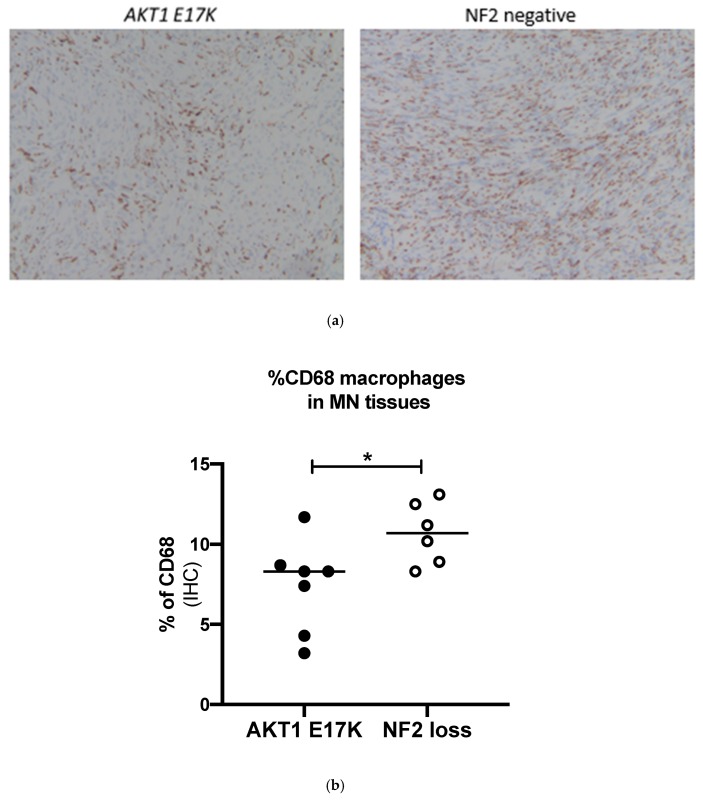
(**a**) Representative immunohistochemistry images for CD68-stained macrophages in *AKT1 E17K-* and NF2-negative meningiomas, ×100 magnification (**b**) % of CD68+ cells in *AKT1 E17K* mutants (*n* = 7) compared to WHO grade I NF2-negative (*n* = 6) meningiomas by IHC; line represents the median, * *p* < 0.05.

**Figure 4:**
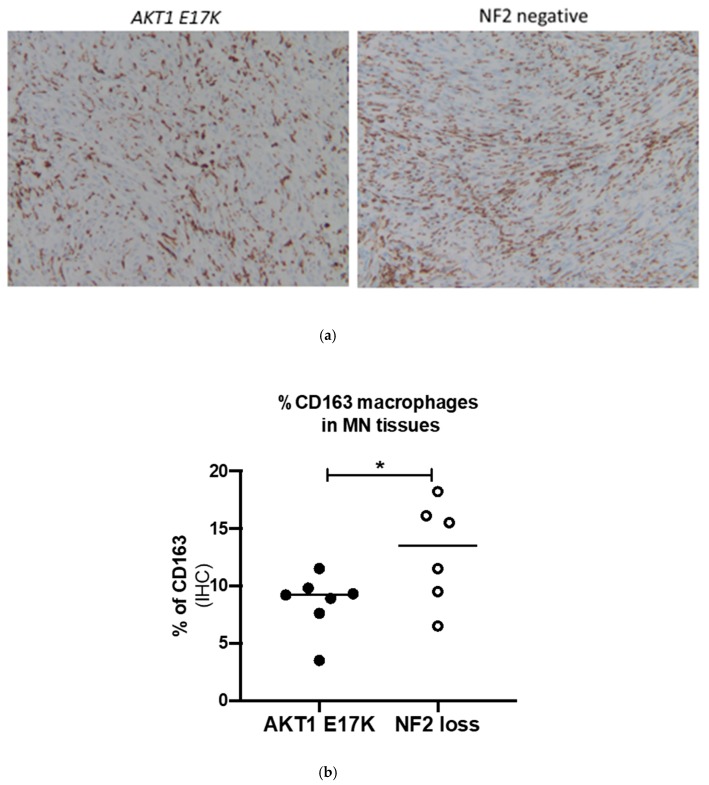
(**a**) Representative immunohistochemistry images for CD163-stained macrophages in *AKT1 E17K-* and NF2-negative meningioma (MN) tissue sections, ×100 magnification (**b**) % of CD163+ cells in *AKT1 E17K* mutants (*n* = 7) compared to WHO grade I NF2-negative (*n* = 6) meningiomas by IHC; line represents the median, * *p* < 0.05.

Comparing the *AKT1 E17K* tumour tissues to the NF2-negative grade I meningiomas showed that *AKT1 E17K* mutants contained significantly fewer CD68 macrophages (7.4 vs. 10.7%; *p* = 0.036; Figure 3) and CD163-positive cells (8.4 vs. 12.9%; *p* = 0.049; Figure 4) compared to NF2 meningiomas, supporting the flow cytometry data suggesting that genotype may influence immune cell filtration over the course of tumour development.

### 2.5. Assessment of Cytokine mRNA Expression Associated with Activated M2 Macrophages

**Figure 5:**
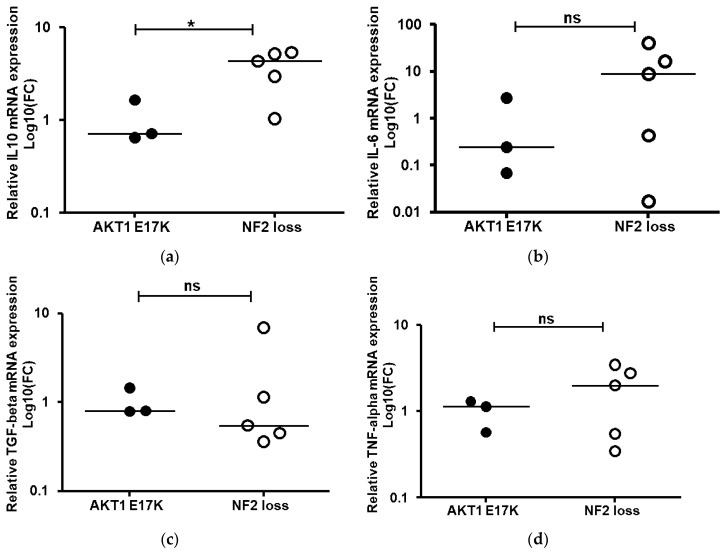
Quantitative real-time PCR (**a**) IL-10, (**b**) IL-6, (**c**) TGF-beta and (**d**) TNF-alpha mRNA expression levels. Relative quantification was obtained using the 2^−^^ΔΔCT^ method normalisation to healthy meninges tissue after using glyceraldehyde-3-phosphate dehydrogenase (GAPDH) as an internal control. Data shown as log 10-fold change (FC) for *AKT1 E17K* (*n* = 3) and WHO grade I NF2-negative (*n* = 5) meningiomas; line represents the median, * *p* < 0.05, ns: not significant.

To better understand whether the higher levels of M2 macrophages observed in the NF2-negative grade I meningiomas were activated, we conducted gene expression analysis of four key cytokines shown to be relevant in M2 macrophage polarization. Comparison of the *AKT1 E17K* and NF2-negative mRNA expression levels determined that IL-10 mRNA was significantly elevated in the grade I NF2 negative meningioma tissues (Figure 5). Collectively, results suggest that M2 macrophages are activated in grade I tumour tissue although, due to the variability in the expression levels for IL-6, TGF-beta and TNF-alpha in individual samples, these results were not significant.

## 3. Discussion

Using our rapid KASP™ genotyping technique and flow cytometry to directly analyse immune infiltrates after tissue dissociation has allowed us to assess larger numbers of tumours quicker and more quantifiably than was previously possible with immunohistochemistry alone.

Loss of NF2 function or loss of heterozygosity at chromosome 22q, where NF2 is located, has been linked to sporadic higher grade meningiomas and poor prognosis [12,38]. The actual effect of NF2 loss on cell populations within a tumour is not known, although the literature does indicate a more immunosuppressive environment in higher grades [21,22,23]. We showed that *AKT1 E17K*-mutated meningiomas have a less immunosuppressive tumour microenvironment when compared to grade I NF2 meningiomas; this may explain why some NF2 tumours have high progression/recurrence rates. The molecular mechanism of how *AKT1 E17K* or NF2 loss influences immunosuppressive environment in the tumour is likely to involve different cell types and be more complex [39]. However, to our knowledge, this is the first time that differences in M2 macrophages in grade I meningiomas have been linked to driver mutations, and could potentially help in developing directed immunotherapies in genetically stratified meningioma patients.

Pinton et al. [22] also observed over 60% lymphocytes (CD45^+^ cells) in fresh tumour cell suspensions using multicolour flow cytometry in 2018, with myeloid cells CD33^+^ HLA-DR^+^ representing the largest population (80.4% +/− 20.6%) of the CD45^+^ group, which was in agreement with our results. Recently, non-progressing grade I meningiomas have been shown to have noticeably higher levels of CD45^+^ inflammatory cells compared to grade II/ III meningiomas [23]. Although these results were not linked to mutational status, they do indicate that immune filtrates play a key role in tumorigenesis, and future detailed studies linking genotype to the tumour microenvironment are warranted.

Variable levels of infiltrating tissue macrophages in meningioma have been previously reported. CD163 is a well-established M2 macrophage marker and has previously been shown using immunohistochemistry to be in high abundance in atypical grade II meningiomas [27,40] but, unlike previous authors, we correlated CD68 (Figure 3) and CD163 expression with NF2 status (Figure 4). Our short-term cell culture data were corroborated by IHC analysis. However, within our dataset we were unable to directly correlate IHC and flow cytometry results for CD163 expression in individual tumours (Supplementary Table S2), which was in agreement with previous literature [41]. Importantly, however, both methods showed that M2 macrophages were higher in NF2-negative tumours by similar relative ratios. Thus, different methods within themselves are consistent. We did not polarise macrophages in our short-term cell culture technique; sample handling may therefore have led to activation of HLA class II of certain populations and could, along with the difference in techniques (surface expression versus cell cross section) explain discrepancies in sensitivity between techniques. Additionally, we used the cell adhesion molecule CD44, previously shown to be expressed on meningioma tumour cells [42,43], to support the large variation in immune filtrates by gating tumour cells (CD45^−^HLA-DR^−^CD14^−^CD44^+^) at each time point.

High CD163 levels have recently been suggested to be a potential biomarker for poor prognosis in a number of solid tumours, including gliomas [44], oestrogen-receptor-positive breast cancer [45] and head and neck squamous cell carcinomas [46]. A major role of macrophages is the clearance of apoptotic cells (efferocytosis), which in the context of cancer is tumour-promoting, inducing a wound-healing immunosuppressive environment. M2 macrophages have a greater capacity for efferocytosis than M1 macrophages, which could explain why higher CD163 levels correlate with poor prognosis in some tumours [47], although, due to the plasticity of tumour-associated macrophages, high M2 macrophage infiltrates do not predict poor overall survival in all cancers [30]. To further support the notion that the CD163-labelled M2 macrophages were activated, we showed expression of IL-10, TNF-α, TGF-β1 and IL-6 in all tumour tissues. Significantly, the key signature cytokine IL-10, known to be produced by all M2 macrophage phenotypes [32], was nearly four-fold higher in the NF2-negative tumours.

Microglia make up 75% of the distinct central nervous system (CNS) immune cell population located in the parenchyma and border areas of the central nervous system. It was previously believed that tumour-associated microglia and tumour-associated bone-derived macrophages exert similar functions in brain tumours; however, recent gene expression analysis in glioblastoma (GBM) revealed that microglia are associated with housekeeping functions such as inducing a pro-inflammatory response, and macrophages are associated with wound healing and immune suppression [48]. Therefore, future work examining the ratio of tumour-associated microglia to macrophages using gene expression signatures in meningiomas and relating this ratio to genotype would be of great interest.

The immune checkpoint marker PD-L1 has been shown to increase in anaplastic meningiomas [49], suggesting that higher-grade tumours harbour a more immunosuppressive tumour microenvironment [50], although immune therapy targeting PD-L1 in meningiomas may be limited due to expression levels [51,52]. Recently, lower proportions of PD-1 positive cytotoxic T cells (CD3^+^CD8^+^FOXP3^−^) have been associated with poorer survival, with longitudinal analyses of the tumour-infiltrating T-lymphocytes suggesting large changes in the microenvironment over the course of the disease [25]. Erkan et al. [53] highlighted in 2019 the importance of an equilibrium between tumour-promoting and anti-immunity pathways in the blood of grade I meningioma patients using a multiplex immunoassay cancer panel, suggesting that progressing grade I meningioma patients may benefit from immunotherapeutic options.

Proctor et al. [51] also recently linked mutations in the PI3K/AKT/mTOR pathway to elevated levels of the immune checkpoint proteins PD-L2 and B7-H3, strengthening the evidence that genetic changes can stimulate a different tumour microenvironment. The impact of somatic alternations on immune responses has previously been shown in BRAF mutations in melanoma [54] and IDH mutations in glioma [55].

In conclusion, although the literature concerning immune cells in meningiomas is less extensive than for other brain tumours such as gliomas, it is highly probable that macrophages play an important role in the growth and progression of meningothelial neoplasms [24]. Future work is required to elucidate the role of M2 macrophages in meningiomas, and to uncover the mechanisms in which tumour cells harbouring a mutation such as *AKT1 E17K* can directly affect the tumour microenvironment. From proteomics studies [36,37] published by our group and others, it is clear that the tumour microenvironment of meningiomas is highly heterogeneous, and that linking cell population, gene expression and/or signalling pathway activation data to genetically defined non-NF2 and NF2 groups would be very beneficial. The simple, cost-effective endpoint genotyping technique used here could aid such stratification in any research or clinical laboratory with a real-time PCR instrument.

## 4. Methods and Materials

### 4.1. Clinical Samples

All subjects gave their informed consent for inclusion before they participated in the study. The study was conducted in accordance with the Declaration of Helsinki, and the protocol was granted full national ethics approval (Approval Date: 20 May 2014) by the South West research ethics committee (REC no: 14/SW/0119; IRAS project ID: 153351), as well as local research and development approval (Plymouth Hospitals NHS Trust: R&D No: 14/P/056 and North Bristol NHS Trust: R&D No: 3458; Approval Date: 18 May 2015). Meningioma MN specimens were collected after planned surgical procedures when surplus tissue was available.

Meningioma SS specimens were collected via UK Brain Archive Information Network (BRAIN UK; https://www.southampton.ac.uk/brainuk/index.page) application “A pilot study analyzing the effect of driver mutations on the (phospho)proteome and microenvironment of meningiomas (Ref no: 15/011)”. BRAIN UK was granted full Health Research Authority approval (Approval Date: 21 July 2015) by the South Central NRES committee (REC no: 14/SC/0098).

### 4.2. End Point Genotyping Method

The samples were cut off excess tumour after diagnostic testing, snap-frozen in liquid nitrogen and then conserved at −20 °C. DNA extraction was performed using the DNeasy kit and blood and tissue miniprep kit (QIAGEN, Manchester, UK), and DNA concentrations were then calculated using Thermofisher Scientific Nanodrop 2000 (Waltham, MA, USA). Endpoint genotyping (Kompetitive Allele Specific PCR (KASP™ [56]) was performed on a Roche Life Sciences Light Cycler 480 II (West Sussex, UK). Oligonucleotides were designed using the genomic reference sequence for each gene. Reactions were carried out in a final volume of 10 μL consisting of genomic DNA (40 ng/μL), 2× KASP low ROX mix and a primer mix containing two allele-specific forward primers and a common reverse primer (Supplementary Table S3; LGC Genomics Ltd., Hertfordshire, UK). Synthetic gene fragments containing known heterozygous or homozygous mutations (gBlocks; Integrated DNA Technologies, Leuven, Belgium) were used as positive controls to aid visualization via Cartesian plots.

### 4.3. Sanger Sequencing

PCR and sequencing primers were designed using the genomic reference sequences and the bioinformatics program Unipro Ugene version 33 (http://ugene.net/download.html). PCR conditions were optimised (Supplementary Table S3) and products were run on 1.2% agarose gels, cleaned up using the Monarch PCR & DNA cleanup kit (New England Biolabs, Ipswich, MN, USA) and sent to Eurofins, Ebersberg, Germany) for Sanger sequencing.

### 4.4. Western Blotting

Cells were lysed in RIPA buffer with protease and phosphatase inhibitors (cocktail tablets, Roche and phosphatase inhibitor cocktail B and C, Santa Cruz Biotechnology, Heidelberg, Germany). Protein concentrations were estimated using the Pierce™ BCA Protein Assay Kit (ThermoFisher Scientific, Waltham, MA, USA), following the instructions of the supplier. Proteins were separated on a 10% Laemmli SDS-PAGE and transferred to a polyvinylidene difluoride membrane (Immun-Blot^®^ PVDF Membrane, Bio-Rad, Watford Hertfordshire, UK). Membrane blocking, antibody incubation and washes were performed as previously described (Supplementary Table S4) [37]. Detection was achieved using the Pierce ECL or ECL Plus Western Blotting substrate (Thermo scientific). Membranes were exposed to Amersham Hyperfilm ECL (GE Healthcare Life Sciences, Amersham, UK). Immunoreactive bands were quantified using Image J software (www.https://imagej.nih.gov/ij/index.html) and each band was normalised to the corresponding GAPDH.

### 4.5. Isolation of Primary Meningioma Cells from Fresh Tissue

Fresh tumour samples were transferred into a sterile tube containing transportation medium (DMEM supplemented with 10% FBS, 500 U/mL penicillin and streptomycin, 2.5 µg/mL amphotericin B). The meningioma were washed twice with sterile PBS and transferred into incubation medium (DMEM supplemented with 10% FBS, 100 U/mL penicillin and streptomycin). Digestion medium consisted of DMEM supplemented with 10% FBS, 100 U/mL penicillin and streptomycin, and 20 units/mL of Type III collagenase (Worthington Biochemical Corp, Lakewood Township, NJ, USA) was added to the plate and incubated at 37 °C overnight. The day after, the meningiomas were mechanically digested and centrifuged at 1500 rpm for 5 min [57]. Cells were seeded into flasks containing meningioma medium (DMEM supplemented with 10% FBS, 1% glutamax, 1% glucose, 100 U/mL penicillin and streptomycin) and incubated at 37 °C in a humidified atmosphere (5% CO_2_). Confluent cultures were split using 0.05% trypsin/EDTA, and medium was changed twice a week.

### 4.6. Flow Cytometry

Primary meningioma cells were washed once in ice-cold flow-staining buffer (PBS, 2% FBS; filter sterilised) and resuspended in flow-staining buffer at a final concentration of 5 × 10^5^ cells. Cells were labelled with the antibodies outlined in Supplementary Table S4 (all from Becton Dickinson (BD) Biosciences, Pharmigen, Wokingham, UK), incubated in the dark at room temperature for 30 min, washed twice and resuspended in ice-cold staining buffer. Relevant single isotype controls were used. Data were collected on 1 × 10^4^ cells per antibody combination on an Accuri flow cytometer (BD Biosciences) and analysis was performed using the Flow Jo software version 10.0 (Lake Oswego, OR, USA).

### 4.7. Immunohistochemistry

Paraffin sections (4 µm) were de-waxed, rehydrated and incubated with primary antibody (Supplementary Table S4) at room temperature O/N after antigen retrieval in Tris/EDTA for 30 min [58]. Proteins were visualised using 3,3′-diaminobenzidine (DAB) and counterstained with haematoxylin (Sigma-Aldrich, Dorset, UK). The immunohistochemical results were reviewed by a neuropathologist (DAH) blinded to the histological grade. Semi-quantitative assessment of staining intensity was assigned as follows: 0 (negative), 1 (low), 2 (moderate) and 3 (strong). Scoring intensity (% of positive cells: a single ×100 image taken and the area fraction stained within this field, also measured using image analysis).

### 4.8. Quantitative Real-Time PCR Analysis

Total RNA was extracted from fresh frozen tissue using the Qiazol^®^ reagent (Qiagen, UK), following the manufacturer’s protocol. The quality and concentration of RNA was established using the NanoDrop 2000) Reverse transcription and real-time PCR was performed on 700 ng of total RNA as previously described [59]. Primers were designed against the reference coding sequence (CDS) for each gene (Supplementary Table S3). *GapDH* gene was used as internal control, all analyses were performed with three replicates and relative gene expression levels were computed using the quantitative 2^−(ΔΔCt)^ method, employing normal meninges tissue as a calibrator [60].

### 4.9. Statistical Analysis

Statistical analysis was performed using the unpaired Student’s t-test performed using GraphPad Prism (https://www.graphpad.com/scientific-software/prism/). A significance level of *p* < 0.05 was considered statistically significant.

## Figures and Tables

**Table 1:** Mutational frequencies detected by endpoint genotyping.

Gene	Mutated/Total	Detected Frequency %	Frequency in Clark et al., 2016 ^b^ %
NF2 (protein loss) ^a^	49/113	43.4	33.4
*AKT1 E17K*	10/159	6.3	10.6
*KLF4 K409Q*	12/158	7.6	7.9
*PIK3CA H1047R*	2/94	2.1	1.5
*POLR2A Q403K*	3/168	1.8	2.5
*SMARCB1 R377H*	1/139	0.7	Not reported
*SMO L412F*	3/150	2.0	3.2

No *PIK3CA E545K (0/95), POLR2A L438-H439del (0/123), SMARCB1 R374Q* (0/140), *SMO W535L* (0/163) mutations were detected in any of the samples screened. ^a^—NF2 loss was assessed by Western blot. ^b^—Expected frequencies taken from 775 meningiomas screened by Clark et al., 2016 [11].

## Supplementary Materials

Supplementary materials can be found at https://www.mdpi.com/1422-0067/21/4/1273/s1. Figure S1—Flow cytometry gating strategy, Table S1—Genotyped samples, Table S2—Macrophage data, Table S3—Olignonucleotide details, Table S4—Antibody details.

## Abbreviations

AKT1	v-akt murine thymona viral oncogene
CD	Cluster of differentiation
GAPDH	glyceraldehyde 3-phosphate dehygrogenase
HLA	Human leukocyte antigen
IHC	Immunohistochemistry
IL	Interleukin
KASP	Kompetitive allele specific PCR
KLF4	Krupple like factor 4
NF2	Neurofibromatosis 2 tumour suppressor gene
PCR	Polymerase Chain Reaction
PIK3CA	Phosphatidylinositol-4,5-Bisphosphate 3-Kinase Catalytic Subunit Alpha
POLR2A	RNA Polymerase II Subunit A
SMARCB1	SW1/SNF-related, matrix-associated, actin-dependent regulator of chromatin subfamily B, member 1.
SMO	Smoothened, frizzled family receptor
TGF-β	Transforming growth factor beta
TNF-α	Tumour necrosis factor alpha
TRAF7	Tumour necrosis factor receptor associated factor 7
WHO	World Health Organisation
