# Supplementary material for: A Rapid Robust Method for Subgrouping Non-NF2 Meningiomas According to Genotype and Detection of Lower Levels of M2 Macrophages in AKT1 E17K Mutated Tumours
Source: Int J Mol Sci. 2020 Feb 13;21(4):1273. doi: 10.3390/ijms21041273 (PMC7073007; doi:10.3390/ijms21041273)
Supplement: Supplementary file 1 [file ijms-21-01273-s001.zip › ijms-687978-supplementary-final/Supplementary figures/Figure S1 - flow cytometry gating strategy.pptx]

## Slide 1
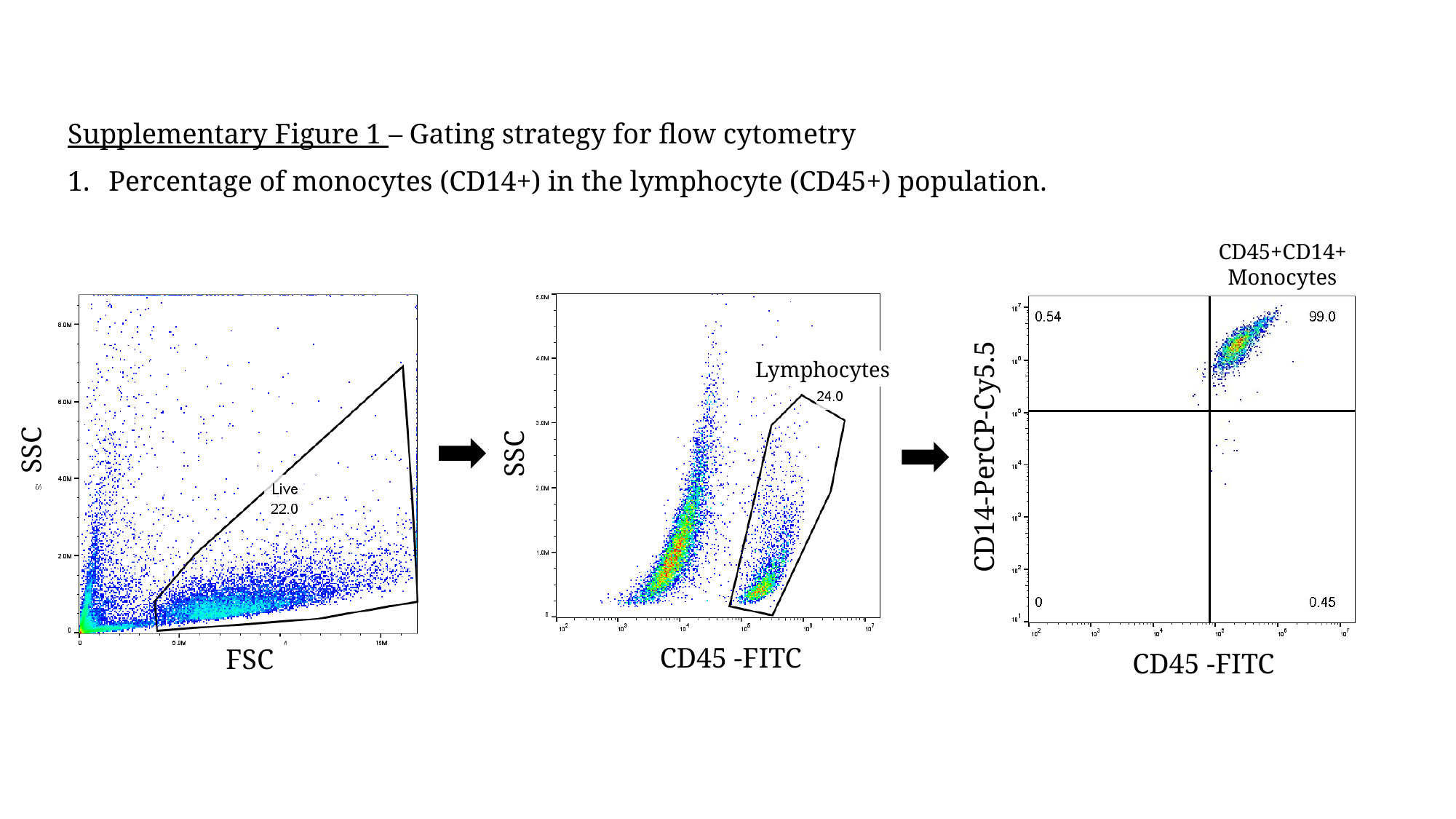

Supplementary Figure 1 – Gating strategy for flow cytometry
Percentage of monocytes (CD14+) in the lymphocyte (CD45+) population.
CD45+CD14+
Monocytes
Lymphocytes
SSC
SSC
CD14-PerCP-Cy5.5
CD45 -FITC
FSC
CD45 -FITC

## Slide 2
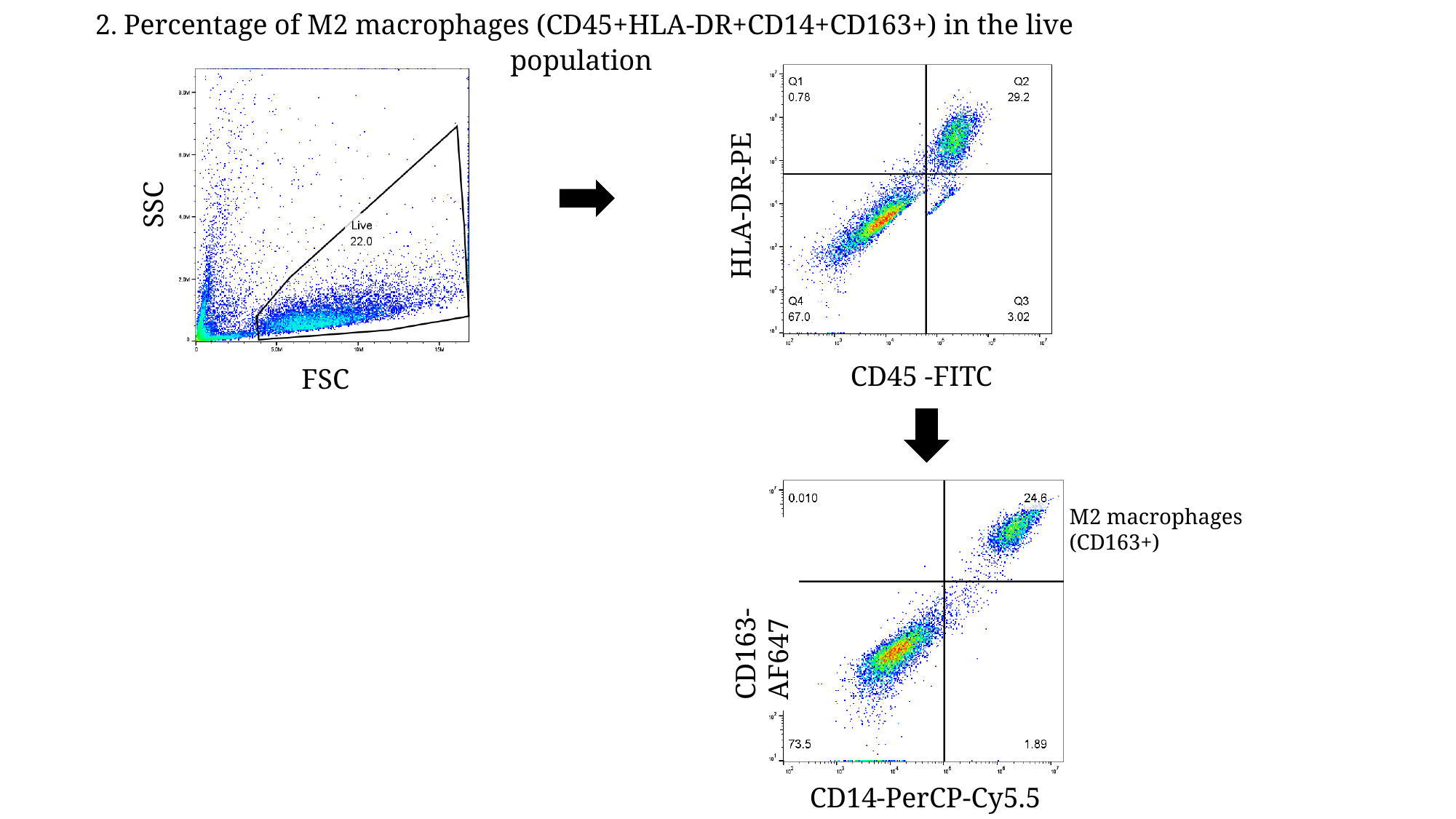

2. Percentage of M2 macrophages (CD45+HLA-DR+CD14+CD163+) in the live population
HLA-DR-PE
CD45 -FITC
SSC
FSC
CD163-AF647
CD14-PerCP-Cy5.5
M2 macrophages
(CD163+)

## Slide 3
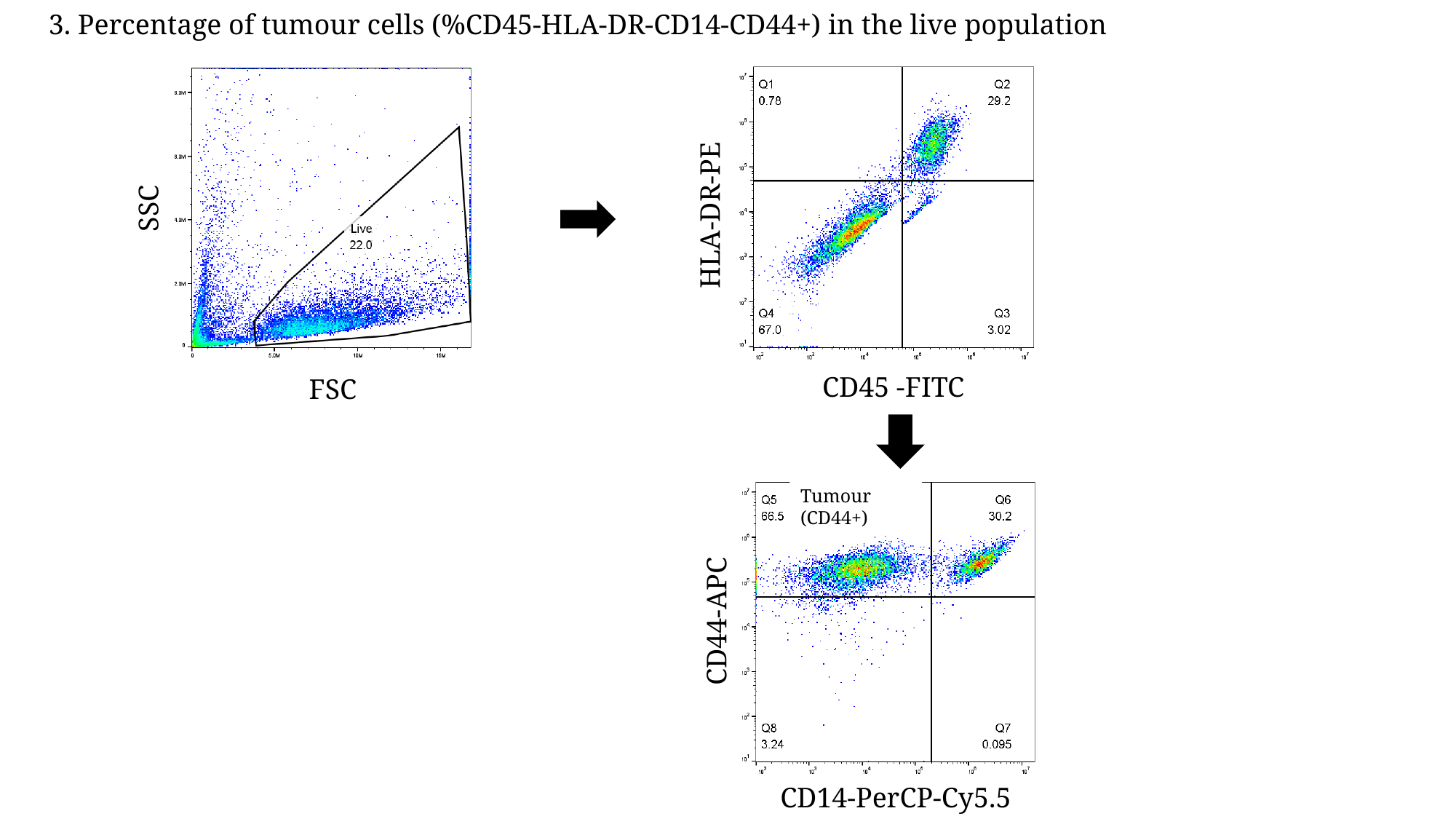

3. Percentage of tumour cells (%CD45-HLA-DR-CD14-CD44+) in the live population
HLA-DR-PE
CD45 -FITC
SSC
FSC
CD44-APC
CD14-PerCP-Cy5.5
Tumour (CD44+)
