# Supplementary material for: A Rapid Robust Method for Subgrouping Non-NF2 Meningiomas According to Genotype and Detection of Lower Levels of M2 Macrophages in AKT1 E17K Mutated Tumours
Source: Int J Mol Sci. 2020 Feb 13;21(4):1273. doi: 10.3390/ijms21041273 (PMC7073007; doi:10.3390/ijms21041273)
Supplement: Supplementary file 1 [file ijms-21-01273-s001.zip › ijms-687978-supplementary-final/Supplementary figures/Table S3. Oligonucleotide details.docx]

**Supplementary Table 3.** Oligonucleotide details

**A.** Endpoint genotyping primers

| WT genotype | GG | AA | AA | CC | GG | CC | AA | GG | ACCTCC | GG | GG |
| --- | --- | --- | --- | --- | --- | --- | --- | --- | --- | --- | --- |
| Allele HEX | G | C | G | T | T | C | G | A |  | G | G |
| Allele FAM | A | A | A | C | G | A | A | G | ACCTTC | A | A |
| Common primer | GTGGCCGCCAGGTCTTGATGTA | GATTACGCGGGCTGCGGCAAAA | GGTAGGAGCCGCTGTACAGGTA | GGGCTTCGTGCTGGCCCCAA | TCCAGATGAGCAGCGTGGCCTT | Not supplied | Not supplied | Not supplied | Not supplied | CATTGCCCTCCCCACTCCTCTT | CCACTCCTCTTCCAGGCGGAT |
| Primer allele HEX (y-axis) | ACCCGCACGTCTGTAGGGG | GTGCCTTGAGATGGGAACTCTG | GAAGGAGCTCACAGGCCTCA | GGAAGTAGCCTCCCACGATGAA | GGCATCGCCATGAGCACCTT | CCTCTTTCCTTCCTAGACTTCAAGAACTACTCCGCCAGGGGG | AAATGAATGATGCACGTCATGGTGGCTGGAC | CTCTCTGAAATCACTAAGCAGGAGAAAGATT | TTTCCACCCCAAGCCCAGTGACCTGCAGACCGGCTATAAG | GTTGGCAAGACGCCTCATCC | GGGGCCGTGTTGGCAAGAC |
| Primer allele FAM (x-axis) | CACCCGCACGTCTGTAGGGA | GTGCCTTGATGGGAACTCTT | AGAAGGAGCTCACAGGCCTCAA | GAAGTAGCCTCCCACGATGAG | GGCATCGCCATGAGCACCTG | CCTCTTTCCTTCCTAGACTTAAAGAACTAGTGCGCAGGGGG | AAATGAATGATGCACATCATGGTGGCTGGAC | CTCTCTGAAATCACTGAGCAGGAGAAAGATT | TTTCCACCCCAAGCCCAGTGACCTTCACCTGCAGACCGGCTATAAG | GTGTTGGCAAGACGCCTCATCT | CGGGGCCGTGTTGGCAAGAT |
| Mutation | *AKT1 E17K* (SNP : (SNP: rs121434592) | *KLF4 K409Q* | *TRAF7 N520S* | *SMO L412F* | *SMO W535L* (SNP : (SNP: rs121918347) | *POLR2A Q403K* | *PIK3CA H1047R* (SNP : rs121913279) | *PIK3CA E345K* (SNP : rs104886003) | *POLR2A L438_H439del* | *SMARCB1 R374Q* | *SMARCB1 R377H (SNP : rs387906812)* |

**B.** Primers used for PCR amplification

| Name | Sequence (from 5’ to 3’) |
| --- | --- |
| AKT1 FOR EXON2 | CTGGCCCTAAGAAACAGCTCC |
| AKT1 REV EXON2 | TGCTTGCCAGCCCAGGACTT |
| TRAF7 R4 FOR | TTTCTGTTGGTGCTGATATTGCCTGGGGGCTGCTTCTCAG |
| TRAF7 R4 REV | ACTTGCCTGTCGCTCTATCTTCCAGAGCCTGTCCACCTATGC |
| KLF4 FOR EXON4 | GGATGATGCTCACCCCAC |
| KLF4 REV INTRON4 | GGAGATCAAGGCGATAGACTGC |

**C**. Primers used for Sanger sequencing

| Name | Sequence (from 5’ to 3’) |
| --- | --- |
| AKT1 FOR SEQ | CACACCCAGTTCCTGCCTGG |
| AKT1 REV SEQ | CGCCACAGAGAAGTTGTTGA |
| TRAF7 WD1 FOR | ATCAACGCGCGGCTGAACAT |
| TRAF7 WD1 REV | GACTCACCCCTGGATGCAGA |
| TRAF7 WD4 FOR | AGCCCACAGTTGCAGCAAT |
| TRAF7 WD4 REV | CTGGCCTTGCACCCAGATG |
| TRAF7 WD5 FOR | ACCAACTGGCCCACGATTAC |
| TRAF7 WD5 REV | GACTTTGGTCTGGTCTGGCG |
| TRAF7 WD6 FOR | CAGTGTCTTTGACCTGCCT |
| TRAF7 WD6 REV | AGCAAGTCCAAACCTGCAG |
| KLF4 FOR SEQ | AGAGGAGCCCAAGCCAAAG |
| KLF4 REV SEQ | TGGGGCTGGAAGCTAACCTG |

**D.** Primers used for the real time PCR

| Name | Sequence (from 5’ to 3’) |
| --- | --- |
| h.GapDH-FOR | ATCACTGCCACCCAGAAGAC |
| h.GapDH-REV | CAGTGAGCTTCCCGTTCAG |
| h.IL10_S168-FOR | CTGCCTAACATGCTTCGAGA |
| h.IL10_AS366-REV | GGTCTTGGTTCTCAGCTTGG |
| h.TGFB1_S1361-FOR | CAGCAACAATTCCTGGCGAT |
| h.TGFB1_AS1562-REV | GGTAGTGAACCCGTTGATGTCC |
| h.il-6_FOR | AGTCCTGATCCAGTTCCTGC |
| h.il-6_REV | AAGCTGCGCAGAATGAGATG |
| h.TNF alpha_S263 -FOR | TGCTTGTTCCTCAGCCTCTT |
| h.TNF alpha_AS461 -REV | GGTTTGCTACAACATGGGCTA |

**E.** PCR conditions

|  | Initial denaturation | Amplification | Final elongation |
| --- | --- | --- | --- |
| *AKT1* and *KLF4* | 98°C for 30 s | 35 cycles :  98°C for 10 s  60°C for 30 s  72°C for 20 s | 72°C for 2 min |
| *TRAF7* | 95°C for 5 min | 25 cycles :  98°C for 10 s  65°C for 15 s  68°C for 4-5 min | 68°C for 5 min |
| IL-6, IL-10, TGF-β, TNF-α | 95°C for 5 min | 45 cycles :  95°C for 10 s  60°C for 20 s  72°C for 10 s | 95°C for 5 min |
