# Supplementary material for: A Rapid Robust Method for Subgrouping Non-NF2 Meningiomas According to Genotype and Detection of Lower Levels of M2 Macrophages in AKT1 E17K Mutated Tumours
Source: Int J Mol Sci. 2020 Feb 13;21(4):1273. doi: 10.3390/ijms21041273 (PMC7073007; doi:10.3390/ijms21041273)
Supplement: Supplementary file 1 [file ijms-21-01273-s001.zip › ijms-687978-supplementary-final/Supplementary figures/Table S4 Antibody details.docx]

**Supplementary Table 4**. Antibody details

A. Flow cytometry antibodies

| **Laser** | **Detection Ab** | **Isotype control** |
| --- | --- | --- |
| FL1 | FITC Mouse Anti-Human CD45  (Clone  HI30; Cat. No.:555482) | FITC Mouse IgG1, κ Isotype Control  (Clone  MOPC-21; Cat. No:555748) |
| FL2 | Anti-HLA-DR PE  (Clone  L243; Cat. No.:347401) | PE Mouse IgG2a, κ Isotype Control  (Clone  G155-178; Cat. No: 555574) |
| FL3 | PerCP-Cy™5.5 Mouse Anti-Human CD14 (Clone  MφP9; Cat. No.: 562692) | PerCP-Cy™5.5 Mouse IgG2b, κ Isotype  (Clone  27-35; Cat. No.: 558304) |
| FL4 | AF647 mouse anti human CD163  (Clone GHI/61; Cat. No.: 562669) | AF647 Mouse IgG1k isotype control  (Clone MOPC-21; Cat. No.:557714) |
| FL4 | APC Mouse Anti-Human CD44  (Clone  G44-26; Cat. No.:560890) | APC Mouse IgG2b κ Isotype Control  (Clone  27-35; Cat. No: 555745) |

B. Western blotting antibodies

| **Primary antibody** | **Species** | **Company** | **Catalogue No.** | **Dilution** |
| --- | --- | --- | --- | --- |
| CD163 (D6U1J) | Rabbit | Cell signalling technology | #93498 | 1:1000 |
| Merlin (D1D8) | Rabbit | Cell signalling technology | #6995 | 1:1000 |
| GAPDH | Mouse | Millipore | MAB374 | 1:20,000 |

| **Secondary antibody** | **Type** | **Company** | **Catalogue No.** | **Dilution** |
| --- | --- | --- | --- | --- |
| Goat anti-Rabbit | HRP-conjugated | Bio-Rad | #170-6516 | 1:10,000 |
| Goat anti-Mouse | HRP-conjugated | Bio-Rad | #172-1019 | 1:10,000 |

C. Immunohistochemistry

| **Antibodies** | **Pretreatment** | **Company** | **Catalogue No.** | **Dilution** |
| --- | --- | --- | --- | --- |
| CD68 | No pre-treatment | Abcam | AB53444 | 1:100 |
| CD86 | 30 min Citrate | Abcam | AB53004 | 1:1000 |
| CD163 | 30 min Citrate | Novocastra | NCL-L-CD163 | 1:1000 |
| CD206 | 30min Citrate | Abcam | AB64693 | 1:1000 |
